# Left ileal conduit: A case report

**DOI:** 10.1002/iju5.12134

**Published:** 2019-12-11

**Authors:** Kazuyoshi Nakamura, Kenichiro Matsui, Ken Wakai, Tomokazu Sazuka, Yusuke Imamura, Shinichi Sakamoto, Nobuyuki Sekita, Tomohiko Ichikawa

**Affiliations:** ^1^ Department of Urology Chiba University Hospital Chiba Japan; ^2^ Department of Urology Funabashi Central Hospital Chiba Japan

**Keywords:** cystectomy, ileal conduit, left abdomen, left side, reverse side

## Abstract

**Introduction:**

When ileal conduit construction is performed for urinary tract drainage during radical cystectomy, the conduit is usually constructed in the right lower abdomen. However, no reports have described ileal conduit construction in the left lower abdomen when it cannot be performed on the right side. In addition, some ingenuity is necessary for construction on the left.

**Case presentation:**

A 75‐year‐old woman visited our hospital with chief complaint of gross hematuria. Computed tomography and cystoscopy showed a huge bladder tumor, and blood analysis showed anemia. The patient was treated by radical cystectomy with ileal conduit construction. An ileal conduit was constructed in the left lower abdomen; it was impossible to construct in the right lower abdomen because of the abdominal wall scar hernia due to the past open surgery.

**Conclusion:**

We herein reported a patient who underwent ileal conduit for urinary diversion on the left side of low abdominal wall.

**Keynote message**

We reported a patient who underwent radical cystectomy followed by ileal conduit on the left side of low abdominal wall. In this case, no perioperative complications, including ileus, were observed after surgery. We herein reported a patient who underwent ileal conduit on the left lower abdomen and described the surgical technique.

## Introduction

Radical cystectomy remains the gold standard procedure for patients with muscle‐invasive bladder cancer.^1^ In Japan, many patients underwent ileal conduit for urinary diversion at many institutions.^2^ In ileal conduit, the stoma opening is generally located in the lower right abdomen. However, whether the conduit can be constructed in the left lower abdomen has not been thoroughly evaluated. There are no reports that have described ileal conduit was created on the left side of the lower abdomen. We herein report a patient who underwent ileal conduit on the left lower abdomen and describe the surgical technique.

## Case presentation

**Figure 1:**
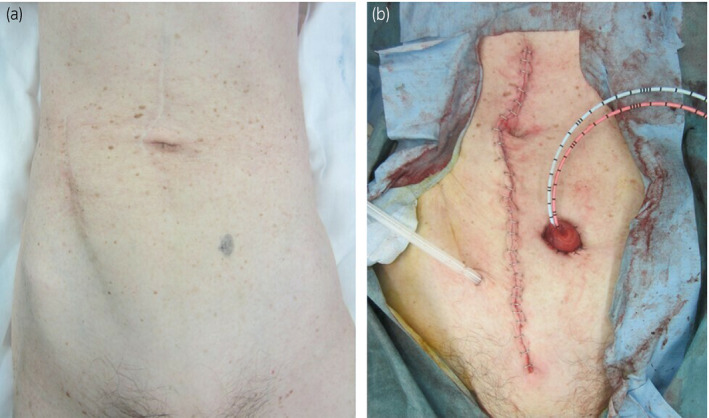
(a) Surgical wound and abdominal wall hernia in the lower right abdomen. (b) Left ileal conduit construction.

A 75‐year‐old woman visited our hospital with chief complaint of gross hematuria. Computed tomography and cystoscopy revealed a huge bladder tumor. Laboratory evaluation revealed a hemoglobin concentration of 7.2 g/dL (normal range 13.7–16.8 g/dL). The patient was treated by radical cystectomy and ileal conduit construction. The patient had a history of open surgery for appendicitis and the presence of an abdominal wall hernia in the surgical wound (Fig. 1a); therefore, construction of an ileal conduit in the right lower abdomen was considered impossible. Thus, we plan to reconstruct an ileal conduit for urinary diversion on the left lower abdomen (Fig. 1b).

**Figure 2:**
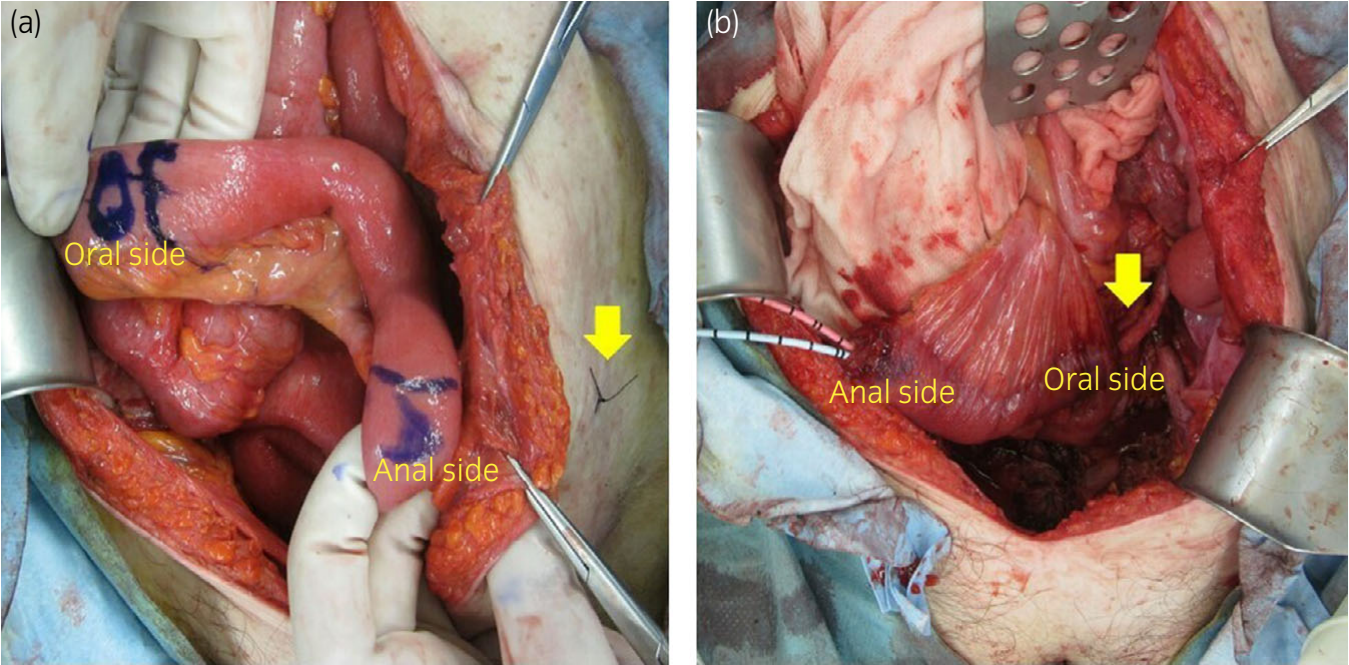
(a) The position at which the anal side of the free ileum could be placed in the left lower abdomen was given top priority, and the distance from the ileocecum was about 55 cm. The arrow indicates the stoma site. (b) The Wallace method was used for ureteral conduit anastomosis.

After radical cystectomy and lymphadenectomy, the right ureter was moved under the sigmoid colon to the left side. In this case, we prioritized the position at which the anal side of the free ileum could be properly placed in the left lower abdomen. The distance from the ileocecum was about 55 cm (Fig. 2a). The ureteral anastomosis was performed on the oral side of the conduit and the stoma was constructed on the anal side so that the intestinal peristalsis was in the same direction as urine flow. The ureteral conduit anastomosis was performed using the Wallace method^3^ (Fig. 2b). Using the peritoneal incision outside the descending colon, the ileal conduit was retroperitoneally formed to cover the ureteral conduit anastomosis. An ileal stoma was then created outside the body through the rectus abdominis muscle.

## Discussion

An ileal conduit is generally constructed in the lower right abdomen. Anatomically, the right side is considered to be the side on which the anal side can properly open to the lower abdomen along the direction of the physiological ileal peristalsis. This may be because the ileal conduit anastomosis readily approaches the ileum regardless of whether the Bricker or Wallace method is used.^4^ Furthermore, it is easy to imagine retroperitonealization of the ileal conduit.

**Figure 3:**
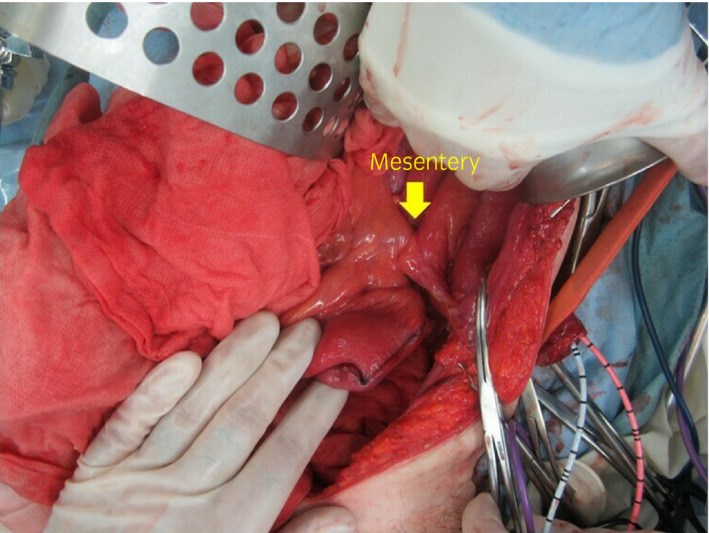
Since the ureteral conduit anastomosis shifts to the head side, attention must be paid to avoid twisting the mesentery in the free ileum.

**Figure 4:**
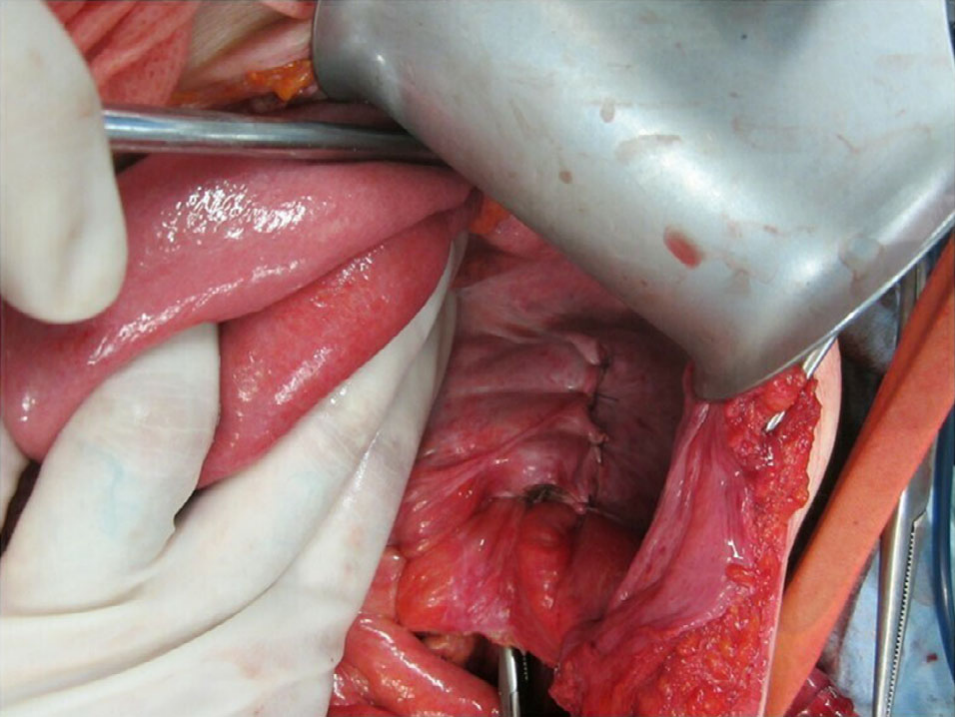
The peritoneal incision is extended outside the descending colon to the cranial side, and the ileal conduit is retroperitonealized.

In contrast, when constructing the conduit on the left side, not only the free ileum preparation site is located far from the ileocecum, but the tube–conduit anastomosis is displaced further toward the head to allow for opening on the anal side to the left lower abdomen along with the peristaltic movement of the ileum. Attention should also be paid to avoid twisting the mesentery of the free ileum (Fig. 3). The peritoneal incision must be extended outside the descending colon to the cranial side to retroperitonealize the ileal conduit, which is displaced more cranially than when construction is performed on the right (Fig. 4). Although not physiological, when the oral side of the ileum is opened to the left lower abdomen, the ureteral anastomosis can be performed at the same height as the right; thus, the operation itself is simple and easy to imagine. Since the urine flow opposes the peristaltic movement of the ileum, there is concern about the conduit ureteral reflux when the ureteral anastomosis is performed using the Wallace method. Even if the Bricker method is used, there is no concern about urinary stagnation in the conduit. However, no reports have described the conduit opening the oral side to the lower abdomen without following the physiological intestinal peristalsis, whether this might be a problem remains unknown. No postoperative bowel complications developed in this case.

## Conclusion

We reported a patient who underwent radical cystectomy followed by ileal conduit on the left side of low abdominal wall. We keep in mind to create ileal conduit on the left side of abdominal wall, if ileal conduit cannot be created on the right side.

## Ethics statement

This case report was approved by the Institutional Review Board of Chiba University Hospital (No. 2564), and written informed consent was obtained from the patient.

## Conflict of interest

The authors declare no conflict of interest.
